# Metabolic shifts in ratio of ucOcn to cOcn toward bone resorption contribute to age-dependent bone loss in male mice

**DOI:** 10.1152/ajpendo.00294.2024

**Published:** 2024-10-23

**Authors:** Matthew Bernhard, Obinna Okorie, Wei-Ju Tseng, Mengcun Chen, Julia Danon, Mingshu Cui, Elisabeth Lashbrooks, Yanmei Yang, Bin Wang

**Affiliations:** ^1^Departments of Medicine, The Center for Translational Medicine, Sidney Kimmel Medical College, Thomas Jefferson University, Philadelphia, Pennsylvania, United States; ^2^Department of Orthopaedic Surgery, Sidney Kimmel Medical College, Thomas Jefferson University, Philadelphia, Pennsylvania, United States

**Keywords:** bone mass, osteoblast, osteocalcin, osteoclast, senile osteoporosis

## Abstract

The study of the senile osteoporosis in men still lags significantly behind that in women. The changes of protein molecule levels and their relationships with bone loss remain poorly understood. In the present study, we used C57BL/6J male mice at ages from 3 to 24 mo to delineate the mechanisms of aging effects on bone loss. We used the microcomputed tomography, mechanical testing, histomorphometry assays, and detection of serum levels of undercarboxylated osteocalcin (ucOcn) and carboxylated osteocalcin (cOcn) to assess bone mass changes and their relationships with the ratios of ucOcn-to-cOcn in mice from different age groups. The results showed that mouse trabecular bone mass reduced gradually with age, whereas cortical bone loss and mechanical property changes mostly occurred in advanced age. Our findings further demonstrated that the increase in osteoclast activity and the decrease in osteoblast function were significantly corelated with blood levels of ucOcn and cOcn, respectively. The dynamic metabolic changes of ucOcn to cOcn ratio were correlated with age-dependent bone loss in mice. In summary, metabolic shifts in the ratio of ucOcn to cOcn toward bone resorption from young adult to elderly mice contribute to the pathogenesis of age-related bone loss. Simultaneously monitoring blood ratios of ucOcn-to-cOcn may be useful to predict the status of bone mass in vivo.

**NEW & NOTEWORTHY** To our knowledge, our finding in this study shows for the first time that metabolic shifts in ratio of ucOcn to cOcn toward bone resorption are markedly correlated with age-dependent bone loss in male mice. These findings for the effects of aging on bone loss will assist in studying the pathogenesis of human type II osteoporosis.

## Graphical abstract

**Figure:**
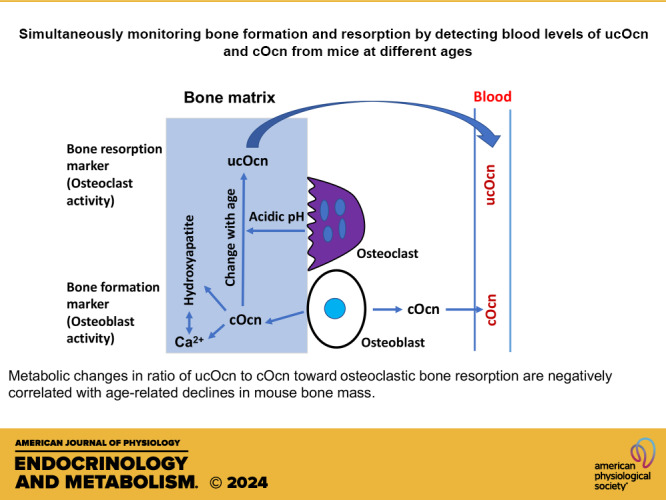

## INTRODUCTION

Osteoporosis is a common metabolic bone disease characterized by decreased bone mass and deterioration of bone microstructure, leading to increased bone fragility and fracture risk (1, 2). Osteoporosis according to the etiology can be divided into primary and secondary osteoporosis. Primary osteoporosis consists of postmenopausal osteoporosis (type I), which occurs in women after stopping menstruation, and senile osteoporosis (type II), which happens in geriatric population in both sexes (3, 4). Secondary osteoporosis is caused by other disorders or medical treatments. Although women are more prone to develop type I osteoporosis, both older men and women are at risk for type II osteoporosis. In contrast to osteoporosis in women, the mechanism of age-related decrease in bone mass in men has been less studied. Multiple factors including androgen deficiency, oxidative stress, and insufficient exercise contribute to the pathogenesis of osteoporosis in men (5, 6). However, the changes of protein molecule levels and their relationships with bone loss remain to be characterized. Given the increasing prevalence of osteoporosis in elderly men, understanding the mechanism of osteoporosis development, which will provide novel strategies to diagnosis and treat osteoporosis, is urgently required.

Bone is a metabolically active organ that undergoes continuous remodeling through the coordinated actions of osteoblastic bone formation and osteoclastic bone resorption (7–9). Osteocalcin (Ocn), whose name is related with calcium and its location in bone (10), contains three gamma-carboxyglutamic acid (Gla) residues and is the most abundant noncollagenous protein in bone. Posttranslational modification in osteoblasts results in the majority of Ocn being completely carboxylated (11, 12) (Supplemental Fig. S1). The carboxylated Ocn (cOcn or Gla-Ocn) is released to the extracellular matrix acting in a paracrine manner (near its site of synthesis) and exhibits high affinity to bind ionized calcium in the extracellular fluid at bone sites, which in turn induces cOcn conformational change and then increases cOcn binding to hydroxyapatite at bone surface to form new bone matrix (13, 14). However, some levels of unbound cOcn can enter the blood circulation, which reflects the osteoblast function. cOcn, therefore, has been implicated as one of bone formation markers (15, 16). During the bone remodeling, cOcn in bone matrix can be converted into a form with a lower or no grade of carboxylation on three glutamate (Glu) residues (undercarboxylated Ocn, ucOcn, or Glu-Ocn), and then be released into the bloodstream when osteoclasts are activated to form acidic pH (10, 11, 17). In contrast to cOcn, ucOcn has less or no affinity to ionized calcium and hydroxyapatite, thereby to be liberated to the bloodstream. The circulating levels of ucOcn are, therefore, dependent on the rate of bone turnover. Osteopetrosis is a rare genetic bone disease characterized by an increase of bone density due to defective osteoclast function (18). Patients with an autosomal dominant form of osteopetrosis were observed to have the reduced ratio of unOcn/cOcn in the bloodstream (18, 19), suggesting a conversion of cOcn to unOcn is decreased when the balance of bone remodeling favors bone formation. In addition, we recently showed that the osteoanabolic drug abaloparatide, a parathyroid hormone-related (1–34) analog, increased the ratio of cOcn/ucOcn in the blood circulation and bone tissue in normal rats (20). In contrast, the ucOcn/total Ocn ratio was higher, whereas the cOcn/total Ocn ratio was lower in men of advanced age (21), and higher ucOcn/total Ocn may also predict fracture risk in older men (22). C57BL/6J mice are often used as models for studying normal aging bone loss (type II osteoporosis) because they share a substantial amount of genetic similarity and low bone density with humans and have a relatively short lifespan (23–25). To date, no studies have attempted to identify the relationship between age-related bone loss and the changes in the ratio of ucOcn to cOcn in male mice.

The aim of this study was to determine whether age-dependent alterations in ratio of ucOcn to cOcn in the bloodstream contribute to bone loss due to the imbalance of bone remodeling in male mice. We used the microcomputed tomography, three-point bending test, histomorphometry assays, and detection of serum ucOcn and cOcn levels to assess bone mass changes and their relationships with the ratios of ucOcn to cOcn in male mice across different ages. Our findings in the present study demonstrate that the dynamic metabolic changes of ucOcn to cOcn ratio are correlated with age-related bone loss in male mice.

## MATERIALS AND METHODS

### Animals

All methods including animal care, blood collection, anesthesia, and euthanasia were performed in accordance with the ARRIVE guidelines. The experimental protocol was reviewed and approved by the Institutional Animal Care and Use Committee (IACUC) of Thomas Jefferson University (Protocol No. 01533). The intact 10 males and 20 female C57BL/6J mice aged 10 wk were purchased from the Jackson Laboratory [(BRID: IMSR_JAX: 000664), Bar Harbor, ME] and maintained in an AAALAC-accredited vivarium (Thomas Jefferson University) for 1 wk before the start of this study. All mice were fed with standard laboratory diets (LabDiet 5001 Rodent Diet, Purina, St. Louis, MO). Food and water were provided ad libitum. Mouse colonies maintained were trio-mated (a single male mouse was placed with two female mice) for production of required experimental animals. Weaning is routinely carried out when litters reach 21–28 days of age. After weaning, male mice were randomly allocated to five groups to grow to 3–24 mo for this study. To estimate the required sample size needed for this study, a power analysis was performed in the experiments. Each group had 14 male mice based on a previous report (26) assessing the significant bone loss in aged mice with 90% power, assuming that a one-way analysis of variance (ANOVA) was done using a significance level of *P* < 0.05. This analysis produced a required sample size of 14 male mice each group, and total 70 mice were used for this study.

To dynamically determine active bone formation at the mineralizing surface, two calcium-binding fluorescent dyes were intraperitoneally injected to mice at two different time points before mouse euthanasia (27). Due to new bone formation related to mouse ages, mice at ages of 3, 6, and 12 mo were injected for calcein (15 mg/kg; Sigma-Aldrich, St. Louis, MO) and alizarin complexone (45 mg/kg; Sigma-Aldrich, St. Louis, MO) at 9 and 2 days before euthanasia, and mice aged 18 and 24 mo received injections of the same dose of calcein and alizarin complexone at 12 and 2 days before euthanasia. After end of mouse ages of 3, 6, 12, 18, and 24 mo respectively, mice were fasted ∼12 h and then anesthetized by using a mixture of 2.5% isoflurane with 100% oxygen for collecting blood via cardiac puncture. Finally, mice were euthanized by carbon dioxide inhalation. After euthanasia, long bones and sixth lumbar vertebra (L6) were collected and cleaned of nonosseous tissues for micro-CT imaging, biomechanical analyses, cryohistology, and histomorphometry by two independent investigators. Lung, kidney, and liver were collected for histological analysis to evaluate any pathological changes. Blood was allowed to clot at room temperature for 30 min and then centrifuged at 1,000 *g* for 15 min to collect serum. Serum was then aliquoted and frozen at −80°C for biochemistry analyses.

### Microcomputed Tomography

The fresh left femurs (LFs) were harvested for high-resolution micro-CT (SKYSCAN 1275, Bruker, Kontich, Belgium). Images were obtained using parameters of 66 KV, 151 μA, pixel size of 7 μm, 7 averages, and a 0.5-mm aluminum filter. Images were then reconstructed using a thresholding of 0–0.055 and beam hardening correction of 20%. The bone parameters were obtained with CTAn v. 1.20.3.0 (Bruker, Kontich, Belgium). Three-dimensional (3-D) renderings were generated using CTVol (v. 2.3.2.0, Bruker, Kontich, Belgium) (28).

### Trabecular Bone Analysis of Left Distal Femurs and L6 Vertebrae

Trabecular bone microstructure was quantified at the distal femurs and in the L6 vertebral body in mice. At the distal femur, a 300-slice thick trabecular volume of interests (VOIs) (corresponding to 2.1 mm thick region), located 120-slice (corresponding to 0.84 mm thick region) proximal to the growth plate, was analyzed. At L6, a 250-slice thick VOI (corresponding to 1.75 mm thick region) was identified at the center of the vertebral body, located at the midpoint between the two endplates. All VOIs were manually contoured to include all trabecular bone and exclude the cortex. A global threshold of 70–255 was used within each VOI. The trabecular parameters, including trabecular bone volume per tissue volume bone volume fraction (BV/TV), trabecular number (Tb.N), trabecular thickness (Tb.Th), and trabecular separation (Tb.Sp) were evaluated. In addition, structure model index (SMI) increase indicates a conversion of trabecular bone from plate-like to rod-like, which is associated with decreased bone strength and increased fracture risk. Connectivity density (Conn.D) indicates the integrity of trabecular bone network and can be negative in trabecular bone evaluation. Both SMI and Conn.D were also evaluated.

### Cortical Bone Analysis and Three-Point Bending Test of Femoral Shaft

Cortical bone microstructure was quantified at the left femur midshaft (middiaphysis) for all mice. At the femur midshaft, standard cortical parameters including cortical thickness (Ct.Th), cortical porosity (Ct.Po), cortical area (Ct.Area), cortical periosteal perimeter (Ct.Ps.Pm), and cortical endosteal perimeter (Ct.Endo.Pm) were evaluated at a 50-slice-thick cortex (corresponding to 0.35 mm thick region) at the center of the femur midshaft. All VOIs were manually contoured to include all cortex and exclude the trabecular bone. A global threshold of 70–255 was used within each VOI.

After the micro-CT scanning, a destructive three-point bending test was performed at the midshaft region with a displacement rate of 0.1 mm/s with the outer supports set 7.5 mm apart (TA Instruments Electroforce 3200, New Castle, DE). The load-displacement curve generated from mechanical testing was used to calculate stiffness, peak load, energy to failure, elastic modulus, toughness, and ultimate stress (29).

### Cryohistology for Dynamic Bone Histomorphometry and Bone Cell Activities

The right tibiae (RT) with fluorochrome label injections were harvested and fixed in 4% paraformaldehyde (PFA) at 4°C for 48 h. Specimens were then transferred to a solution of 20% sucrose and 2% polyvinylpyrrolidone (PVP) for 48 h, followed by cryoembedding in Tissue-Tek O.C.T. Compound (Sakura Finetek USA Inc., Torrance, CA). The sectioning, staining, and imaging methods were referred to previous publications (30, 31). About 8 μm thick mineralized coronal plane sections using a Leica RM2155 microtome were collected using cryofilm IIC tape (SECTION-LAB Co. Ltd., Hiroshima, Japan). Sections were attached to glass microscope slides using 1% chitosan adhesive for 48 h and were then rehydrated in a 1× phosphate-buffered saline (PBS) solution for 15 min and mounted with 50% glycerol. Each section in proximal tibiae was subjected to three rounds of imaging on the EVOS M7000 Imaging System (Thermo Fisher Scientific Inc, Waltham, MA) including *1*) tibial bright-field image and mineralization label using bright-field, GFP, and Texas Red emission filter channels to obtain calcein and alizarin fluorescent images; *2*) tartrate-resistant acid phosphatase (TRAP) enzymatic activity staining (ELF97 yellow fluorescent acid phosphatase substrate, Thermo Fisher Scientific Inc, Waltham, MA) using a customized ELF97 emission filter channel (32); and *3*) alkaline phosphatase (AP) enzymatic activity staining (Vector Blue Alkaline Phosphatase Substrate kit, Vector Laboratories) using Cy5 emission filter channel. After the imaging of multiple rounds was selected as the region of interest with 1 mm below the primary spongiosa, bone formation sites were identified by fluorochrome double labels on the bone surface. Bone formation and bone resorption surface were examined by AP staining and TRAP staining on the bone surface, respectively. Standard dynamic histomorphometric parameters, including mineralizing surface per bone surface (MS/BS), mineral apposition rate (MAR), and bone formation rate per bone surface (BFR/BS) in trabecular bone of tibiae were calculated using OsteoMeasure (OsteoMetrics, Atlanta, GA) according to the ASBMR Histomorphometry for Nomenclature Committee (33). Bone formation surface (AP.S/BS) for osteoblast activity and bone resorption surface (TRAP.S/BS) for osteoclast activity in trabecular bone were also obtained using OsteoMeasure.

### Serum Biochemistry

The levels of cOcn and ucOcn from mouse serum in each group were quantified by using mouse Gla-Ocn (cOcn, Cat. No. MK127, RRID: AB_2847843, Takara, Japan) highly sensitive EIA kit and mouse Glu-Ocn (ucOcn, Cat. No. MK129, RRID: AB_3626017, Takara, Japan) highly sensitive EIA kit Glu-Ocn, respectively according to the manufacturers’ instructions. cOcn EIA kit uses a mouse Ocn carboxy-terminus specific antibody as a capture antibody on a solid-phase plate. An enzyme-labeled antibody specific to cOcn (N-terminus amino acids) is used as the detection antibody. ucOcn EIA kit also uses a mouse Ocn carboxy-terminus specific antibody as a capture antibody on a solid-phase plate. A monoclonal antibody that is specific to the Glu residues that straddle positions 17 and 20 of mouse Ocn is arranged as the detection antibody. Thus, these two kits can be used together for simultaneous detection of cOcn and ucOcn levels. A standard curve was plotted based on the results obtained from the standard solutions, and the curve was used to determine the corresponding concentrations of cOcn or ucOcn based on the sample’s absorbance. The concentrations of each sample were then multiplied by the dilution ratio and expressed as nanograms per mL.

### Pathological Changes in Lung, Kidney, and Liver

After collection of blood and bone, we then harvested lung, kidney, and liver to observe whether there were possible tumor and pathological changes for hematoxylin-eosin (H&E) staining in these tissues from different age groups especially aged mice. We only found one mouse with lung tumor in 24 mo old, and this mouse was excluded in the study.

### Statistics

All statistical analyses were performed using GraphPad Prism v. 9.4.1 for Windows [GraphPad Software (RRID: SCR_002798), San Diego, CA]. Results in the figures are presented as bar graphs with dot plots, which maximize visualization of the data distribution. For the animal study, *n* indicates the mouse number in the experiments. A one-way ANOVA with a Tukey honestly significant difference (HSD) post hoc test was performed to determine the treatment aging effects among groups. The correlations between study variables were performed by using Spearman’s rank correlation analysis. For all analyses, *P* < 0.05 was considered to indicate statistical significance.

## RESULTS

### Mouse Trabecular Bone Mass Gradually Reduces with Age

Bone mineral density is directly associated with the body weight. We first recorded mouse body weight in 3, 6, 12, 18, and 24 mo old before mice were euthanized. We found that male mouse body weight increased with age (Supplemental Fig. S2). The greatest body weights were in ages between 12 and 18 mo (∼32 g). The body weight then declined significantly at the age of 24 mo (28.4 g).

**Figure 1.**
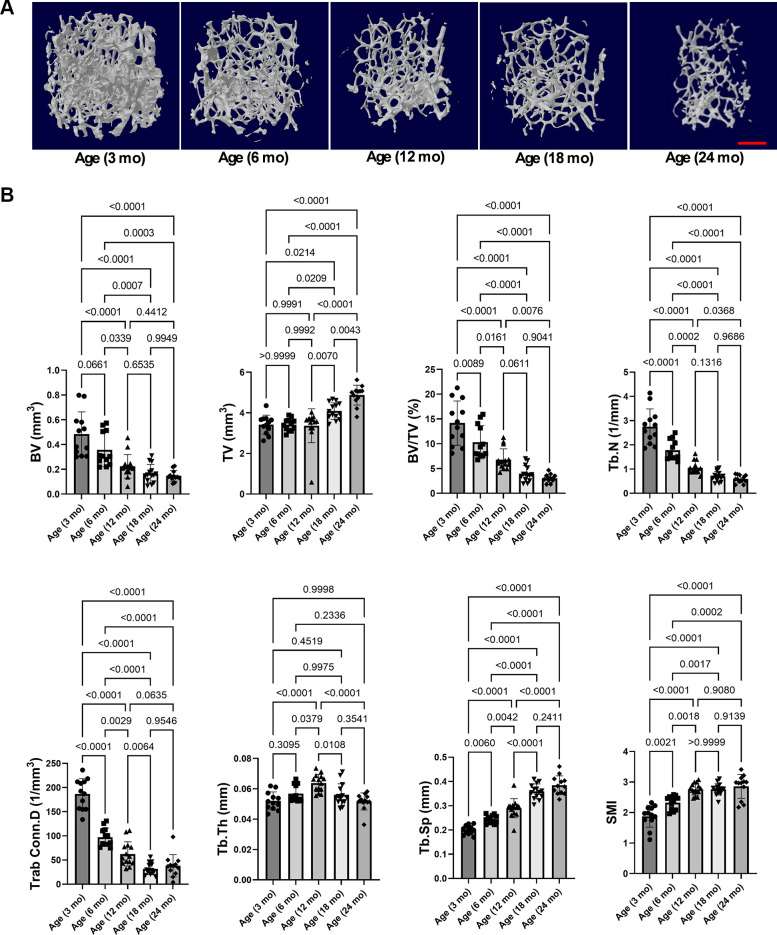
Mouse trabecular bone mass gradually decreased with age. *A*: representative 3-D renderings of the distal femur from mice at different ages. *B*: after micro-CT, the parameters of microstructure changes were analyzed. Comparisons between groups in trabecular bone microstructure of distal femur from mice at different ages. *n* = 12–14. Results were presented as bar graphs with dot plots. *P* < 0.05 was considered significant. Scale bar: 500 µm. 3-D, three-dimensional; BV/TV, bone volume/total volume; Conn.D, connectivity density; mo, month; SMI, structure model index; Tb.N, trabecular number; Tb.Sp, trabecular separation; Tb.Th: trabecular thickness.

Bones are composed of two macroscopic forms: trabecular bones (also known as cancellous or spongy bones) and cortical bones (compact bone). We next assessed microstructure changes at the trabecular bone in distal femur from mice at different ages. Trabecular bone volume was the highest for young adult mice and diminished steadily thereafter. The trabecular bone volume decreased by 26% between 3 and 6 mo, volume decreased by 37% with significance between 6 and 12 mo, volume decreased by 26% between 12 and 18 mo, and volume decreased by 11% between 18 and 24 mo. As mouse age increased, bone volume fraction (BV/TV), trabecular number (Tb.N), and connectivity density (Conn.D) were significantly decreased, whereas trabecular separation (Tb.Sp) and structure model index (SMI) significantly increased (Fig. 1). Trabecular thickness (Tb.Th) was maintained and then declines slightly in advanced age, which is consistent with previous report in male mice (26). Overall, mouse trabecular bone mass gradually reduced with age.

We also assessed the microstructure changes of trabecular bone in L6 from mice at different ages. Likewise, as mouse age increased, BV, BV/TV, Tb.N, connectivity density (Conn.D) significantly diminished, whereas Tb.Sp and SMI were significantly increased (Supplemental Fig. S3). There was a trend toward decline in Tb.Th, especially after mouse age at 12 mo. Collectively, trabecular bone mass was the highest for young adult mice at 3 mo old, and the loss of trabecular bone in both appendicular (i.e., distal femur) and axial (i.e., L6) sites exhibited in an age-dependent manner.

### Mouse Cortical Bone Loss Occurs Only in Advanced Age

**Figure 2.**
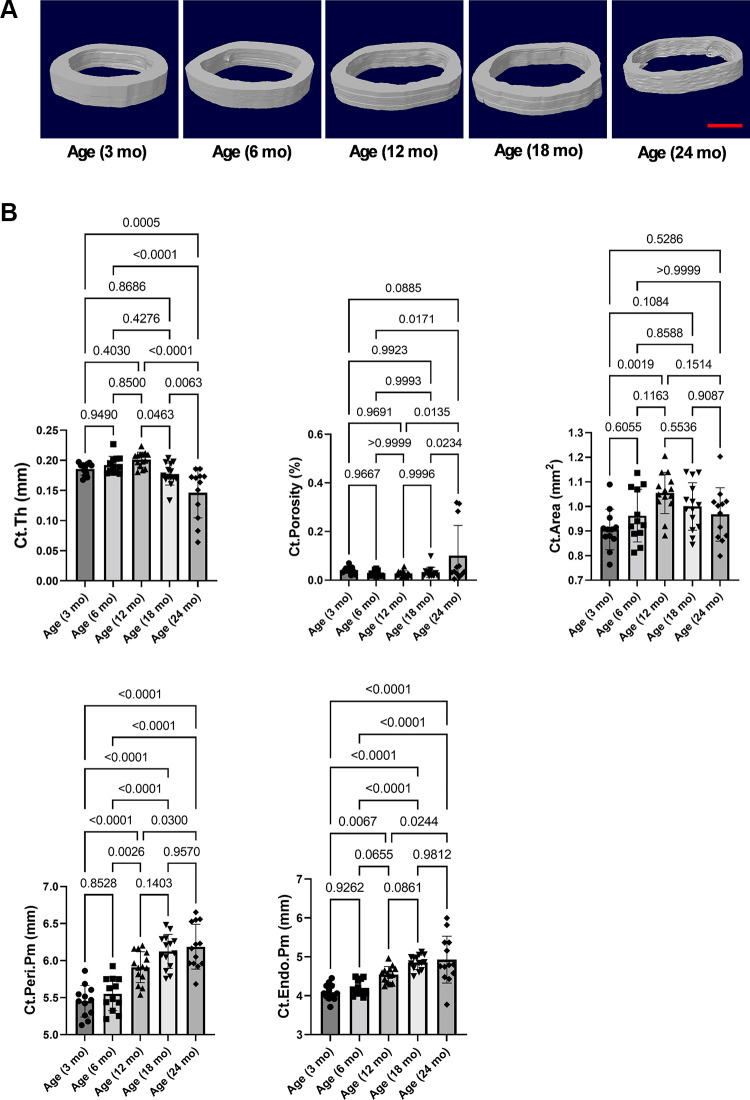
Mouse cortical bone loss occurs only in advanced age. *A*: representative 3-D renderings of femur midshaft from mice at different ages. *B*: after micro-CT, the parameters of microstructure changes were analyzed. Comparisons between groups in cortical bone microstructure of femur midshaft from mice at different ages. *n* = 12–14. Results were presented as bar graphs with dot plots. *P* < 0.05 was considered significant. 3. Scale bar: 500 µm. 3-D, three-dimensional; Ct.Area, cortical area; Ct.Endo.Pm, cortical endosteal perimeter; Ct.Po, cortical porosity; Ct.Ps.Pm: cortical periosteal perimeter; Ct.Th: cortical thickness.

We then assessed microstructure changes in cortical bone in mice at different ages. Midshaft of long bone is a common site for evaluating cortical bone mass. At femur midshaft, there were no significant changes for cortical thickness (Ct.Th) and cortical porosity (Ct.Po) between 3 and 18 mo, but these parameters were significantly reduced between 18 and 24 mo (Fig. 2). There was a trend toward decline in cortical area (Ct.Area) between 12 and 24 mo. The cortical endosteal perimeter (Ct.Endo.Pm) increased with age, which supports the increased osteoclastic activity at the cortical endosteal area. In addition, cortical periosteal perimeter (Ct.Peri.Pm) also increased with age, suggesting continuous cortical bone growth. Together, in contrast with trabecular bone loss, mouse cortical bone loss occurs only in advanced age.

### Mouse Mechanical Property Changes Mostly Occur in Advanced Age

**Figure 3.**
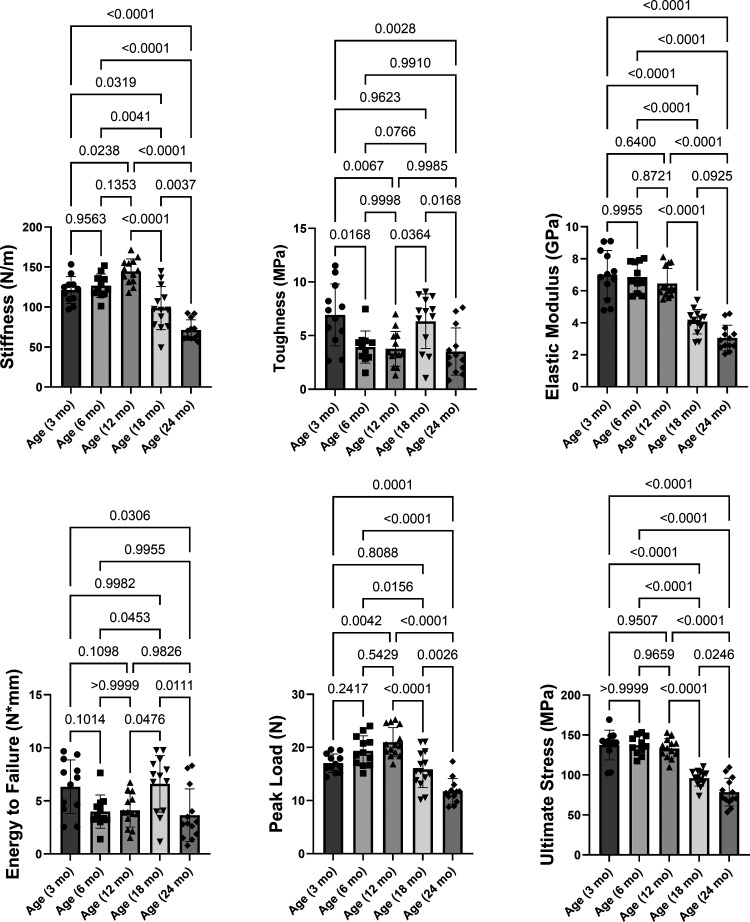
Mouse mechanical property changes mostly occur in advanced age. A destructive three-point bending test was performed at the midshaft region with a displacement rate of 0.1 mm/s with the outer supports 7.5 mm apart. Comparisons between groups in cortical bone microstructure of femur midshaft from mice at different ages. *n* = 12–14. Results were presented as bar graphs with dot plots. *P* < 0.05 was considered significant.

Cortical bone microstructure change contributes to the bone strength. In the femur midshaft as whole bone mechanical properties, the changes of stiffness, elastic modulus, peak load, and ultimate stress started from 12 mo of mice (Fig. 3). All parameters of mechanical properties were significantly reduced between 18 and 24 mo, indicating bone strength became worse in advanced age. In addition, we found that Ct.Th was positively correlated with the changes in stiffness, elastic modulus, peak load, and ultimate stress (Supplemental Fig. S4).

### Mouse Histomorphometric Changes at Different Ages and Imbalance of Bone Remodeling toward Osteoclast Bone Resorption

**Figure 4.**
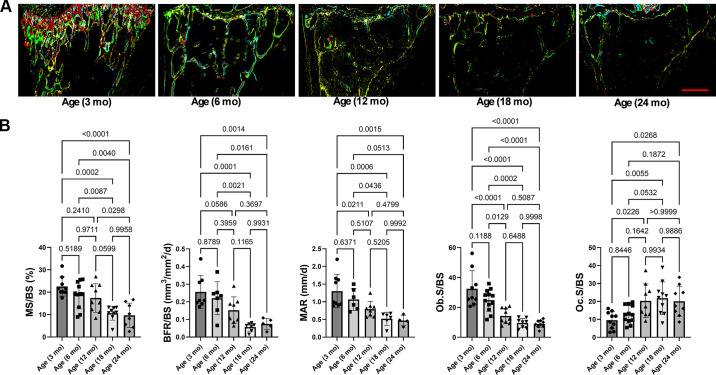
Mouse histomorphometric changes at different ages and imbalance of bone remodeling toward osteoclast bone resorption. *A*: representative images of mice at different ages with overlaid multiplexed cryohistological imaging of calcein in green, alizarin complexion in red, AP staining at the bone surface in teal, and TRAP staining at the bone surface in yellow. *B*: dynamic bone histomorphometry of treatment groups of osteoblast activities (Ob.S/BS), osteoclast activities (Oc.S/BS), MS/BS, BFR/BS, and MAR. Results were presented as bar graphs with dot plots. *P* < 0.05 was considered significant. Scale bar: 500 µm. AP, alkaline phosphatase; BFR/BS, bone formation rate per bone surface; MAR, mineral apposition rate; MS/BS, mineralizing surface per bone surface; TRAP, tartrate-resistant acid phosphatase.

To test whether age-dependent bone loss is attributed to changes of the osteoblast and osteoclast activities, we conducted multiple rounds of staining and imaging on the same cryosection to determine the relationships between fluorochrome labeling and bone cell enzymatic activities (30, 31). We first determined histomorphometric changes in trabecular bone at the proximal tibia from mice at different ages (Supplemental Fig. S5). There was a trend toward decrease in mineralizing surface per bone surface (MS/BS), mineral apposition rate (MAR), bone formation rate per bone surface (BFR/BS) between 3 and 12 mo. After 12 mo old, these parameters were significantly reduced compared with those in mice at 3 mo old (Fig. 4). We further found that bone formation enzymatic activity of alkaline phosphatase per bone surface (AP.S/BS) was gradually reduced to 24% from 6 mo and reached 43% reduction with significance at the age of 12 mo. Importantly, the bone resorption enzymatic activity of tartrate-resistant acid phosphatase per bone surface (TRAP.S/BS) increased gradually with age and reached to significant increase with 59% in mice at the age of 12 mo old. Taken together, these data indicate that bone remodeling skewed toward osteoclastic bone resorption during aging.

### Distinct Blood Levels of ucOcn and cOcn Display in Mice among Different Age Groups

**Figure 5.**
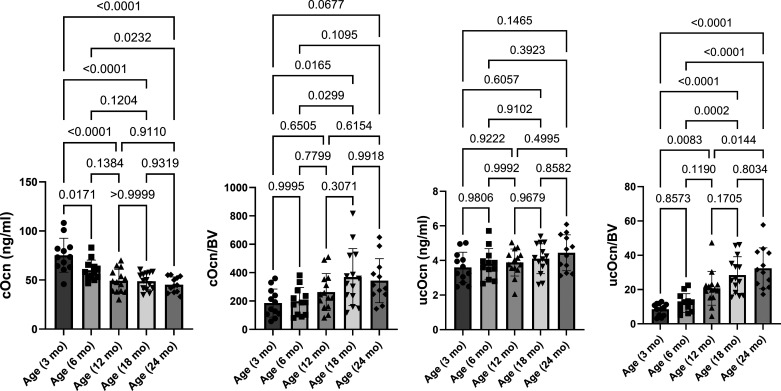
Distinct blood levels of ucOcn and cOcn display in mice at different ages. Blood levels of ucOcn, cOcn were measured in mice among the different age groups. The adjustments of ucOcn and cOcn with bone volume (BV) were calculated. Results were presented as bar graphs with dot plots. *P* < 0.05 was considered significant. cOcn, carboxylated osteocalcin; ucOcn, undercarboxylated osteocalcin.

Since bone is a metabolically active organ, we explored whether any protein molecule levels in the bloodstream were changed in mice at five different ages. Previously, total blood Ocn levels were recognized as a bone formation marker (34). Total Ocn has been identified to comprise both cOcn and ucOcn, which represent osteoblast and osteoclast functions, respectively. We found that blood levels of cOcn decreased gradually with age (Fig. 5) and reached significant reduction in mice aged 6 mo old, which was consistent with osteoblastic activity (AP.S/BS). We also found that there was a trend toward increase in blood levels of ucOcn during aging. However, when ucOcn levels were adjusted to bone volume (BV) at different ages, the values of ucOcn/BV increased significantly with age, which was consistent with osteoclastic activity (TRAP.S/BS) (Fig. 5).

### Metabolic Changes of ucOcn to cOcn Ratio Are Correlated with Age-Dependent Bone Loss

**Figure 6.**
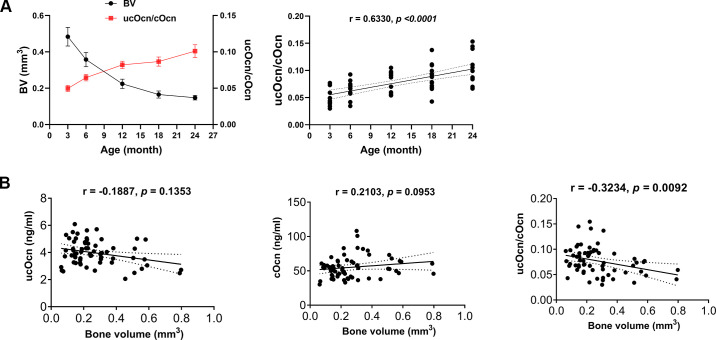
Metabolic changes of ucOcn to cOcn ratio are correlated with age-dependent bone loss. *A*: the ratios of unOcn to cOcn increased with age, whereas bone volume decreased over life. *B*: the correlation of blood levels of ucOcn and cOcn, or the ratio of ucOcn to cOcn with bone volume (BV) in mice among different age groups were conducted by using Spearman’s rank correlation analysis, respectively. Data are presented as means ± SE. *P* < 0.05 was considered significant. cOcn, carboxylated osteocalcin; ucOcn, undercarboxylated osteocalcin.

Bone volume is an important parameter for bone quantity, reflecting the property of bone mass. We found that bone volume decreased with age, whereas ratios of unOcn to cOcn increased in aging mice (Fig. 6*A*). To test whether blood levels of ucOcn and cOcn or ratio of ucOcn to cOcn can be used to predict the status of bone mass, we first conducted the correlation of ucOcn, cOcn, and ratio of ucOcn to cOcn with trabecular bone volume, respectively. We found that unOcn was negatively corelated with bone volume, suggesting that osteoclast function increases with age (Fig. 6*B*). We also found that cOcn was positively corelated with bone volume, suggesting that osteoblast function reduced with age. Importantly, the ratio of ucOcn to cOcn negatively corelated with bone volume significantly, suggesting that blood ratios of ucOcn to cOcn can be used to predict the status of trabecular bone mass in mice at different ages.

We also conducted the correlations of blood levels of ucOcn, cOcn, and ratio of ucOcn to cOcn with cortical bone porosity, cortical bone thickness, and other parameters. No significant correlations were found between blood levels of ucOcn, cOcn, or ratio of ucOcn to cOcn with cortical bone parameters (data not shown) because mouse cortical bone loss occurs only in advanced age.

## DISCUSSION

Understanding of the impact of aging on bone loss in male mice will help study the type II osteoporosis in men. In the present study, we used C57BL/6J male mice among the different age groups (26, 35), in attempt to elucidate the molecular mechanism of the effects of aging on bone loss. Five different ages from 3- to 24-mo-old mice represent young adult, mature adult, and elderly mice (36). Our findings demonstrated that mouse trabecular bone mass in both femur and lumbar vertebra was highest in 3 mo and then bone loss displayed in an age-dependent way. This contrasts with previous reports that mice of C57BL/6J and other inbred strains did not undergo bone loss by 12 mo of age, a discrepancy that may be attributed to the use of different detecting methods (26, 37). However, mouse cortical bone loss and mechanical property changes did not occur in young and mature adult mice but happened only in advanced age, consistent with site-specific bone loss reported in both humans and mice (26, 38, 39). In addition, mouse body weights were significantly reduced at the age of 24 mo. The cortical bone makes up nearly 80% of the total skeletal mass, and bone minerals may contribute to body weight. Our data indicated that the cortical bone of the femurs became markedly porous, and this likely contributes to be the decrease in total body weight in 24-mo-old mice (40).

Osteoporosis is a metabolic bone disorder, and bone loss in aged mice results from enhanced osteoclast-mediated bone resorption and insufficient osteoblast-driven bone formation (41). cOcn is produced by osteoblasts in bone and its level in the bloodstream represents osteoblast function. In contrast, ucOcn reflects the function of osteoclasts, which can produce acidic pH to convert cOcn to ucOcn in the bone matrix, the latter of which does not bind to hydroxyapatite at bone surface and then is released to the circulation. Our data showed that the blood levels of cOcn were reduced gradually with age, which is consistent with the bone volume reduction. In contrast to cOcn, the blood levels of ucOcn were not reduced but slightly increased with age. After adjustment to reduction of bone volume (BV), the ratio of ucOcn/BV significantly increased with age. These findings further confirmed that the bone remodeling balance skewed toward osteoclast bone resorption with aging. In addition, we found that the ratios of ucOcn to cOcn increased with age and negatively correlated with age-related trabecular bone loss, and this correlation did not occur in aged-related cortical bone loss. This discrepancy may be ascribed to the different structures of trabecular and cortical bone, with trabecular bone exhibiting greater metabolic activity due to its larger surface area.

The carboxylation at three glutamic acid residues requires γ-glutamyl carboxylase and vitamin K functions as a cofactor for this enzyme. Previous studies (20, 42) showed that addition of vitamin K to osteoblast cultures increased cOcn secretion but reduced ucOcn formation, whereas warfarin, a competitive inhibition of the vitamin K epoxide reductase, reduced cOcn but increased ucOcn production. Whether the increase in blood levels of ucOcn in older mice was due to a reduction in vitamin K intake cannot be excluded and should be explored in future studies.

Osteoporosis is considered a “silent disease,” and clinical diagnosis of osteoporosis relies largely on bone density scanning. However, radiographically detectable changes in bone mass can lag months to over a year behind. Bone biomarkers, which are released during bone remodeling by actions of osteoblasts or osteoclasts, can offer prognostic information that supplements radiographic assessment of bone mass (15, 43). Blood total Ocn was used for many years as a marker of bone formation in human and animal studies. Since the different functions of ucOcn and cOcn were recognized, detection of blood total Ocn (ucOcn plus cOcn), could, therefore, enable it to become two contradictory markers. Total Ocn has not been used since 2011 when the International Osteoporosis Foundation and the International Federation of Clinical Chemistry and Laboratory Medicine recommended that the procollagen type I N-terminal propeptide (P1NP) be used as a marker of formation and the carboxyl‐terminal cross linking telopeptide of type I collagen I (CTX-1) as a marker of resorption in clinical studies on osteoporosis (34, 44). It has been accepted that cOcn is a marker of osteogenesis, whereas ucOcn is a marker for bone resorption. However, the normal rangers of blood levels of ucOcn and cOcn are not consistent in humans and animals from the published data (14, 20, 45–48). These differences may be attributed to the different methods for detecting blood levels of ucOcn and cOcn. One widely used method is according to their diverse binding affinities with hydroxyapatite (49). Thus, cOcn amounts are calculated by subtracting unOcn from the total Ocn amount. Since the enzyme-linked immunosorbent assay (ELISA) kits for detection of human, rat and mouse ucOcn and cOcn are commercially available, many laboratories have already used these kits for detecting blood levels of ucOcn and cOcn (14, 50). In the present study, we used mouse ELISA kits to detect blood levels of ucOcn and cOcn in mice at different ages. Although more animal samples are needed to confirm our finding, simultaneously measuring blood ucOcn and cOcn levels may help predict the bone formation and resorption status or assess the efficacy of drug treatment on bone mass in vivo (20, 22, 51).

Bone has been recognized as an endocrine organ (52). Bone-derived ucOcn is considered another hormone with manyfold functions, including regulation of glucose metabolism and male fertility (19, 53–55). It has been reported that ucOcn released into systemic circulation binds to GPRC6A (12, 56, 57), one of G protein-coupled receptors, on the cell surface of pancreas and testes to regulate insulin and testosterone secretion. The latter effect of testosterone may lead to increase in estradiol (5, 58). Published data have demonstrated that ucOcn improves glucose metabolism and insulin sensitivity, and that both testosterone and estradiol counteract osteoblast apoptosis but stimulate osteoclast apoptosis (5, 19, 55). This raises the issue that if ucOcn is beneficial for energy metabolism and stimulates testosterone and subsequent estradiol secretion, why does bone loss still occur in aged mice? In 2020, researchers from two laboratories independently found that Ocn was not physiologically involved in glucose metabolism and testosterone synthesis in new Ocn knockout mice (14, 59). These findings are inconsistent with data from the original Ocn knockout mice (60). Therefore, there are some limitations of the current study that we did not analyze the causal correlation between the blood levels of ucOcn with glucose, testosterone, or estradiol. In addition, gonadectomy was not performed in this study, and the effects of glucose metabolism and sex hormones on bone loss in male mice at different ages were not examined. Thus, the notions for ucOcn regulation of glucose metabolism and sex hormone formation in male mice among different age groups need to be examined in Ocn and/or GPRC6A knockout mice (14, 59, 61).

In summary, age-related bone loss in C57BL/6J male mice occurs in site-specific manner, with trabecular bone loss progressing at a higher rate than cortical bone. Blood levels of ucOcn and cOcn are correlated with osteoclast and osteoblast activities, respectively, in aging mice. Metabolic shifts in the ratio of ucOcn to cOcn from young adult to elderly mice support the notion that bone remodeling during aging skews the balance of bone remodeling to favor osteoclastic bone resorption. Although our data need to be confirmed in other male strains and female mice, our findings for the effects of aging on bone loss will assist in studying the pathogenesis of human type II osteoporosis. In addition, our findings highlight the potential utility of dynamically simultaneous detections of unOcn and cOcn to predict the bone formation and resorption status in vivo. In this regard, before the normal rangers of blood levels of ucOcn and cOcn at different ages in various species are determined, a large number of blood samples need to be studied in the physiological and pathological conditions in future bone studies.

## DATA AVAILABILITY

All data that support the findings of this study are available from the corresponding author upon reasonable request.

## SUPPLEMENTAL MATERIAL

10.6084/m9.figshare.27095845 Supplemental Figs. S1–S5: https://doi.org/10.6084/m9.figshare.27095845.

## GRANTS

This work was supported by the National Institutes of Health (NIH) Grants R01-DK-119280, R01-AR077666, and R01-AG-071025 (to B.W.). Analysis of three-pointing bending was supported by Penn Center for Musculoskeletal Diseases’ NIH Grant P30-AR-069619.

## DISCLOSURES

No conflicts of interest, financial or otherwise, are declared by the authors.

## AUTHOR CONTRIBUTIONS

B.W. and M.B. conceived and designed research; M.B., O.O., W.-J.T., M.C., J.D., M.C., E.L., and Y.Y. performed experiments; M.B., O.O., W.-J.T., M.C., J.D., M.C., E.L., Y.Y., and B.W. analyzed data; M.B., O.O., W.-J.T., M.C., and B.W. interpreted results of experiments; M.B., O.O., and M.C. prepared figures; M.B. and B.W. drafted manuscript; M.B., W.-J.T., M.C., and B.W. edited and revised manuscript; M.B., O.O., W.-J.T., M.C., J.D., M.C., E.L., Y.Y., and B.W. approved final version of manuscript.
